# Availability of best practices for opioid use disorder in jails and related training and resource needs: findings from a national interview study of jails in heavily impacted counties in the U.S.

**DOI:** 10.1186/s40352-022-00197-3

**Published:** 2022-12-20

**Authors:** Christy K. Scott, Christine E. Grella, Michael L. Dennis, John Carnevale, Robin LaVallee

**Affiliations:** 1 Chestnut Health Systems, 221 W. Walton St, Chicago, IL 60610 USA; 2 Chestnut Health Systems, 448 Wylie Dr, Normal, IL 61761 USA; 3 Carnevale Associates LLC, 4 Belinder Rd, Gaithersburg, MD 20878 USA

**Keywords:** Jail, Opioid use disorder (OUD), Opioid overdose, Best practices, Medication for opioid use disorder (MOUD), Re-entry services, Training needs

## Abstract

**Background:**

Jails are optimal settings in which to screen individuals for opioid use disorders (OUD) and provide needed services, especially medications for OUD (MOUD). This study sought to assess the availability of OUD “best practices” in jails located in counties heavily impacted by opioid overdose in the U.S. and their related training and resource needs. Counties were selected for study inclusion using two indicators of OUD severity: the absolute number and population rate of opioid overdose deaths. Structured interviews were completed with representatives from 185/244 (76%) of targeted counties and 185/250 (74%) of targeted jails in these counties. Ten OUD best practices were identified based on current treatment and practice guidelines. These include: screening for OUD; clinical assessment; medically managed withdrawal; MOUD administration; MOUD for pregnant people; counseling and wrap-around services; collaboration with community providers; assistance with Medicaid/insurance; re-entry services; and overdose prevention. Descriptive analyses examined the provision of any services and average percentage of services endorsed within best-practice categories, association of best-practice availability with community and jail characteristics, and related needs for training and resources.

**Results:**

Over 70% of jail respondents indicated that some aspects of each of the ten OUD best practices were available within their jails, ranging from 71% using clinical assessment to 96% providing overdose prevention. However, there was considerable variability in the average percentage of items endorsed within each best-practice category, ranging from 38% of items regarding re-entry services to 88% of items regarding medically managed withdrawal. Availability of OUD best practices in jails also varied by community and jail characteristics. Jails reported the highest needs for funding for medication and clinical staff.

**Conclusions:**

Policies are needed to address the identified gaps in availability of OUD best practices within jails. Training, technical assistance, and funding are needed to improve clinical capacity of jails to administer MOUD and to ensure continuity of care from jail to community, which are essential to reducing the risk of opioid-related overdose following release.

**Supplementary Information:**

The online version contains supplementary material available at 10.1186/s40352-022-00197-3.

It is widely recognized that individuals with opioid use disorders (OUD) come into frequent contact with the criminal justice system (Chen et al., 2022; Winkelman et al., 2018), making the interface between these individuals and jails a unique opportunity to intervene and change the trajectories of both OUD and criminal justice system involvement. A recent study of one county found that approximately one fifth of all overdose deaths occurred among individuals who had been incarcerated in the county jail within the prior 2 years, with each booking increasing the risk of fatal overdose by about 20% (Victor et al., 2022). Moreover, the rates of fatal and non-fatal opioid overdose have escalated in the past 20 years (Hedegaard et al., 2020) and continue to increase (Mattson et al., 2021; https://www.cdc.gov/drugoverdose/nonfatal/dashboard/index.html), making it imperative that services to address OUD are available to individuals who come into contact with carceral settings.

Given the ample evidence that individuals with OUD are at high risk of opioid-related fatality upon their release from a period of incarceration (Binswanger et al., 2007; Binswanger et al., 2013; Merrall et al., 2010), many local jurisdictions have recognized the need to address the treatment of individuals with OUD while in their custody. Prominent professional organizations have also recently released policy reports and toolkits to promote the implementation of MOUD and Naloxone within jails and post-release (see: GAINS Center for Behavioral Health and Justice Transformation & Substance Abuse and Mental Health Services Administration [SAMHSA], 2020; National Governors Association & American Correctional Association, 2021; National Sheriffs’ Association & National Commission on Correctional Health Care, 2018; Substance Abuse and Mental Health Services Administration (SAMHSA), 2019). Moreover, there have been several successful legal challenges finding that the failure to provide MOUD to individuals while incarcerated violates the Americans with Disabilities Act, is medical malpractice, and constitutes cruel and unusual punishment (American Civil Liberties Union, 2021; Brezel et al., 2020).

A growing body of research demonstrates the effectiveness of treatment with MOUD for justice-involved populations (Moore et al., 2019) as it relates to reducing the risks of recidivism (Evans et al., 2019) and opioid-related relapse, overdose, and mortality (Evans et al., 2022; Lee et al., 2016). In addition, treatment with MOUD while detained or incarcerated is associated with greater likelihood of continued treatment following discharge (Hass et al., 2021; McKenzie et al., 2012; Sharma et al., 2016). Yet jails face numerous barriers to providing services to address OUD. These include stigma regarding people with OUD and OUD treatment. Negative attitudes about people with OUD among the general population may inhibit expansion of and funding for MOUD treatment, particularly for justice-involved populations. A national population survey of beliefs about opioid addiction found that stigma associated with OUD was positively associated with support for discriminatory actions against people with OUD in areas such as education, health care, employment, and housing; it was negatively associated with support for expanding insurance coverage for treatment of OUD, expanding access to Naloxone, and increasing government funding for OUD treatment (Adams et al., 2021; Taylor et al., 2021).

Among correctional administrators and staff, use of MOUD is often considered inferior to abstinence-based treatments and viewed negatively as a “substitute” addiction, leading to policies that restrict its use (Streisel, 2018; Wakeman & Rich, 2018). Moreover, many individuals with OUD have had prior aversive experiences in jails, such as being forced to undergo withdrawal if currently on MOUD or receiving inadequate medication dosing, leading to negative beliefs about use of MOUD (Fu et al., 2013; Rich et al., 2015). Administrative barriers also limit MOUD availability in jails, including those related to licensing and regulations on dispensing of medications; other barriers are concerns about diversion and limited space and clinical capacity (Friedmann et al., 2012; Grella et al., 2020). A recent study of MOUD implementation in four prison and jail systems in the Northeast underscored barriers to implementing MOUD stemming from a lack of funding and clinical space; negative perceptions related to MOUD; frequent exclusions or discontinuation of treatment based on patient factors, movement or transfer of individuals; and challenges to sustaining care coordination at the time of release (Ferguson et al., 2019).

Even when MOUD and related services are reported as being *available* within jails, access to and actual provision of these medications within jails are often severely restricted in terms of who can receive it and when. For example, in some jails where MOUD has been nominally available, its use has often been restricted to pregnant women, and usually discontinued at the end of the pregnancy (Fiscella et al., 2004; Sufrin et al., 2020). Not only is access to MOUD limited to specific subgroups of individuals, access can be limited in terms of when someone can actually receive it, for example, only upon release. One study using administrative records found that slightly less than one third of individuals who were screened as having OUD received any MOUD while incarcerated (Ray et al., 2022). This is a good example of jails reporting that MOUD is available but not disclosing that its actual availability is significantly restricted, leading to inaccurate characterizations of the extent of MOUD accessibility.

Although prior studies have examined the availability of MOUD in jails as well as the barriers to its delivery, much less is known about the incorporation of best practices for addressing OUD among individuals in jail and the training, resource, and technical assistance needs to expanding the provision of best practices to this population. This study uses the framework of the OUD service cascade, which has been used to evaluate the sequential process by which individuals are screened and engage in treatment for OUD, particularly MOUD, and to identify gaps in service delivery (Scott et al., 2020; Socías et al., 2018; Williams et al., 2017; Williams et al., 2018). This study was guided by the following research questions:

1. To what extent have OUD best practices been incorporated in jails that are located in areas that have been most heavily impacted by opioid-related overdose?
2. Does availability of OUD best practices vary across characteristics of the jails and communities in which they are located?
3. What types of training, technical assistance, and resources are needed to expand the availability of best practices for OUD in jails?

## Methods

### Jail selection

Given the lack of county-level prevalence data on OUD, the research team used opioid overdose deaths as a proxy for the opioid epidemic severity in counties. Opioid overdose deaths are not evenly distributed across the general population or counties, with the vast majority of overdose-related mortality concentrated in a sub-set of counties. Thus a representative sample of jails would have large standard errors and the average would primarily reflect the practices of jails in counties with relatively low rates of opioid-related mortality – and therefore may have less imperative or ability to respond. A second issue was that the epidemic looks very different in urban and rural areas (Altekruse et al., 2020; Haffajee et al., 2019; Hollingsworth et al., 2017; Monnat, 2018, 2019; Pear & Monnat, 2019; Rigg et al., 2018). Therefore, this study focuses on the counties with the highest concentrations of opioid-overdose mortality. In consultation with senior scientists from the Bureau of Justice Statistics and a nationally recognized sampling statistician, two strategies were used to identify a census of counties that were highly impacted by opioid-related deaths: 1) the subset of counties that accounted for half of all opioid-related deaths, and 2) those counties that had a significantly higher rate of opioid-related deaths per 100,000 people than the national average.

Using data from the Centers for Disease Control and Prevention Multiple Causes of Death, the research team identified 48,476 opioid-overdose deaths in the U.S. during 2017. This represented the most recent annual data available just prior to initiation of the study, comprising surveys of state prison systems (see Scott, Dennis, Grella, Mischel, & Carnevale, 2021) and jails (present study). Supplement A contains two maps showing, by county, the number of opioid-overdose deaths and crude rates of opioid overdose death in 2017. While there is overlap in the counties, the two maps show distinct patterns: high numbers of opioid-overdose deaths predominate in counties with large urban populations, whereas high rates of opioid-related deaths occur in more rural counties. Those meeting both criteria (*n* = 56) tend to be more mid-size counties. Taken together, the two maps show how the opioid epidemic is concentrated in a subset of all U.S. counties and varies by region.

To derive the study sample of counties, we identified the top 97 counties that accounted for 50% of all opioid overdose deaths nationally. Second, we identified an additional 147 counties that had crude rates of opioid-related overdose deaths per 100,000 people with their 95% confidence interval completely above that for the U.S. rate (14.5 to 14.8). The combined census of 244 counties accounted for 66% of all opioid-related deaths in the U.S. and had significantly higher crude rates of opioid-related overdose deaths per 100,000 people than the U.S. overall (20.3 vs. 14.7). These counties were diverse in both total population and jail census. In terms of population, 14% of the counties had 1 million or more people, 36% had 250,000 to 999,999 people, and 50% had fewer than 250,000 people. In terms of the number of people confined to jail, 11% of the counties had jails with 5000 or more people confined, 41% had 1000 to 4999 people confined, and 48% had less than 1000 people confined.

To identify the jails within each of the above counties, the research team used information from the Bureau of Justice Statistics and National Institute of Corrections. Additional information was obtained from internet searches of county websites, searching for “jail,” “detention center,” “sheriff’s office,” or “sheriff’s department” in each county to obtain the name and address of the sampled jail and phone number and/or email address for an office or person to use as an initial contact. Consistent with earlier studies (Foudray et al., 2021), these jails included public and privately run facilities, other confinement facilities (e.g., detention centers), and facilities with individuals that were both pre- and post-adjudication. While most counties had only one primary jail under a Sheriff or county administrator, the list included some large city jails, regional jails serving more than one county, and large urban jails that held people from other nearby jurisdictions. We excluded other community supervision programs (e.g., diversion, electronic monitoring, house arrest, probation, parole) and temporary holding facilities only used by courts or police for 72 hours or less. We also excluded counties from states with a single integrated prison-jail system that were included in a prior report (Scott, Dennis, Grella, Mischel, & Carnevale, 2021).

### Measurement

#### Interview data

The interview was structured as a survey to assess availability and accessibility of: 1) opioid withdrawal management; 2) screening and assessment to identify opioid use problems; 3) MOUD provision, including eligibility, reasons for use, and restrictions on use; and 4) re-entry planning and services, consistent with other criminal justice-focused service cascade models (Belenko et al., 2017; Dennis et al., 2019; Ray et al., 2022). Given the high risk of opioid overdose during incarceration and following release, we included a section on overdose prevention services, based on recommendations of the National Commission on Correctional Health Care (2022). Lastly, respondents were asked about their needs for training, technical assistance, and resources to improve their ability to provide OUD services.

Study measures were developed through a review of previous studies of OUD-related services and treatment within both prisons and jails, as well as consultation with correctional representatives and stakeholders. Information on interview development, including the interview measures and the sources consulted to derive the interview content, are contained in Supplement B. To aid in evaluating the quality of the responses when the information was not available, the interview’s survey format allowed respondents to indicate whether their response was estimated, or whether the information was not accessible to them or was not collected. Most questions were closed-ended, although “other” responses and open-ended questions were included where relevant. These responses were keyed verbatim.

#### Geocode data

To help understand the patterns of OUD service delivery in jails, additional county-level geocoded data were linked to the interview data to examine community characteristics, such as gender and racial and ethnic demographics, population rate of incarceration, urbanicity, level of poverty in the county in which the jail was located, and availability of MOUD providers in the county. Some of these data are represented as average percentages of population characteristics across counties and others are mean values. These data came from the Opioid Environment Policy Scan database at geodacenter.github.io/opioid-policy-scan/. This public use data set includes data from the Bureau of Justice Statistics, Centers for Disease Control and Prevention, the Current Population Survey, and SAMHSA (Kolak et al., 2021).

### Procedures

The study team collaborated with state and local sheriff’s associations and other key stakeholders in the targeted counties to identify appropriate respondents for the interview. An interview coach was assigned to each jail contact to provide a study overview, answer questions, and facilitate the interview process. Coaches were BA- or MA-level research assistants with prior experience working on criminal justice-related studies/projects and were extensively trained through videos, webinars, in-person training and review of digital recordings. All interviews were attempted between December 2019 to February 2021, and the interview focused on the most recent 12-month period. For all jails, this 12-month referent period occurred prior to onset of the COVID-19 pandemic.

The structured interview protocols were in a “survey” format. Typically, no individual could answer all the questions and several required consulting other resources, such as annual reports or other administrative data. Hence, the interview process was conducted through a series of stages: a) approaching individual jails through state or other professional associations they trusted; b) providing them an overview of the study’s purpose, survey component, options for completing it, and the coaching process; c) agreeing on the best way for each individual jail to respond via interview, written response or a combination of both (95% of jails opted for a combination); at this time, administrators often designated an individual or a multidisciplinary team to complete the interview; d) answering questions during this process by their coach; e) returning the instrument via phone, email, fax, or a combination of the three; this sometimes included review of the draft answers with back and forth follow-up to clarify any inconsistencies and explore ways to fill in any missing data; and f) providing each jail with a summary report of their answers vs the answers of all jails across the country and answering any questions they had (in a few select cases, correcting an answer). Only then was the data for each jail considered final.

The interview process required four to twelve weeks to finalize and cumulative interview time ranged from 30 to 90 minutes. Depending on the range of services provided, as indicated by the respondent and availability of information requested, most interviews required input from multiple people/sources and often took place over several sittings to collect the requested information. Phone interviews were audio-recorded to ensure the accuracy of responses. All interviews were reviewed for completeness, inconsistent responses, and legibility.

Interviews were completed with representatives from 185/244 (76%) of targeted counties and 185/250 (74%) of the targeted jails in these counties, which is comparable to or higher than prior surveys conducted with correctional administrators (Taxman et al., 2007) or that use online or mail contact only (Foudray et al., 2021). Additional detail on respondent characteristics is provided in the Results section. Respondents were not compensated for their participation. The study obtained a Certificate of Confidentiality from the National Institutes of Health and was conducted under the supervision of Chestnut Health Systems’ Institutional Review Board for the Protection of Human Subjects.

### Analyses

Data were analyzed with IBM SPSS™ Version 27, using the frequencies and descriptive procedures, including t-tests for continuous measures and chi-square tests for categorical measures. Responses were eliminated from the analyses when data for 15% of counties on any given item were missing. This includes when it was either unavailable or not systematically collected. Where one or more counties were missing, the mean of the valid responses was reported (the equivalent of mean replacement). For some questions (e.g., type of MOUD available), there were skip outs, and the questions were asked only when applicable. These cases are clearly noted in the text and tables.

The primary unit of analysis is a “jail” at the organizational level. Although the interview included some questions that asked respondents to report characteristics and services at the individual (detainee) level, these data were often unavailable to the jail administrator or could not be easily retrieved. Because of the large amount of missing information, data from these questions at the individual-level were eliminated from analyses.

### OUD best practices

To evaluate the availability of best practices to address OUD within jails, the study team reviewed treatment and practice guidelines that recommended best practices for developing, implementing, and sustaining jail-based OUD-related services (see Supplement B). Best practices were recommended by a variety of sources, including treatment protocols endorsed by SAMHSA for withdrawal management, induction and maintenance treatment with MOUD, and ongoing monitoring for the general population (2018) and specifically for criminal justice settings (2019); and guidelines and recommendations from the National Sheriffs’ Association and National Commission on Correctional Health Care (2018), and the National Governor’s Association and American Correctional Association (2021). These documents recommended best practices that were based on recommendations from expert panels, review of best practices that were not criminal justice specific (e.g., American Society of Addiction Medicine), and systematic literature reviews and other scientific literature (e.g., Grella et al., 2021). Review of these materials led to identification of ten core OUD best practice domains that were consistently addressed across these sources, following the general framework of the OUD service cascade within correctional settings. These included: 1) screening for OUD, 2) clinical assessment by qualified treatment provider, 3) medically managed withdrawal, 4) MOUD administration, 5) services for pregnant women, 6) counseling and wrap-around services, 7) collaborative relations with community MOUD providers, 8) assistance with applications for state Medicaid/insurance coverage, 9) re-entry services, and 10) overdose prevention.

Between 1 to 7 items from the interview that corresponded to each of the 10 OUD best-practice domains are listed individually in the descriptive statistics. We also calculated the average number of items endorsed across each domain. Finally, a summative score was computed for percentage of services provided across the 10 best-practice categories (possible range 0–100%, median = 67%), which was used to categorize jails into 2 groups based on a median split: Low (0–67% of items endorsed), and High (above 67%). The association of best-practice categories (Low, High) with community and jail characteristics using the geocoded data was compared using crosstabulations and chi square statistics for categorical variables and t-tests for mean values – with jail as the unit of observation and analysis.

## Findings

### Jail and respondent characteristics

Survey responses were obtained from 185/244 (76%) of targeted counties and 185/250 (74%) of the targeted jails in these counties. To evaluate whether there was non-response bias, we compared jails that did and did not complete the interview using geocoded data on characteristics of the jails and the communities in which they were located (tabled results in Supplement C). Respondent jails were significantly (*p* < .05) more likely than non-respondents to be from counties that have larger populations (Means = 575,523 vs. 517,011). Relative to jails that responded, those that did not respond were more likely to be from counties with more people living below the federal poverty line (13% vs. 15%), and to have higher population incarceration rates per 100,000 people overall (324 vs. 409) as well as specifically for females (97 vs. 129), males (555 vs. 699), and white non-Hispanics (245 vs. 317). There were no significant differences between responding and non-responding jails by census region, urbancity, population race/ethnicity, jail size, or availability of MOUD providers within the county.

Among the study sample, nearly all jails (95%) are under the auspices of the Sheriff’s office or the county. Most jails house both males and females (96%), although 4% house males only. When asked about the model of health care services provision, 72% indicate they contract out for services, 11% use direct services provision, and 16% use a hybrid or other type of model. The average number of admissions across participating jails during the 12-month reference period of 2019 was 17,232 (*SD* = 57,710; total = 3,187,920 admissions), although this was highly variable with a median of 6602 and a range of 545 to 636,833.

The respondents for the 185 jails included jail administrators (54%), medical/behavioral health directors (18%), health services administrators (8%), program/service directors (6%), and other administrative staff (14%). They reported an average of 5.0 years (*SD* = 4.9) in their current position and 16.0 years (*SD* = 10.8) in the corrections field. In most cases, respondents identified one (*n* = 101), two (*n* = 43), or three (*n* = 2) additional people who assisted in gathering needed information. These additional respondents were jail administrators (31%), health services administrators (22%), medical/behavioral health directors (15%) or providers (13%), and program/service directors (12%) who had an average of 5.1 years (*SD* = 5.4) in their current position and 14.2 years (*SD* = 10.6) in the corrections field.

### Availability of OUD best practices in jails

Table 1 shows the distribution of the ten OUD best practice categories and their respective sub-items for the total sample. Over 70% of respondents indicate that provision of some aspects of each of the ten best practices are available within their jail, ranging from 71% of jails providing clinical assessment to 96% providing overdose prevention. However, there is considerably more variability in the extent to which services are provided based on average percentage of items endorsed within each category, ranging from 38% of items regarding re-entry services to 88% of items for medically managed withdrawal.

**Table 1:** Evidence-Based Opioid Use Disorder Best Practices in a Sample of U.S. Jails (*N* = 185)

OUD Best Practice Category (average of activities)/ Specific Activity	% of jails
**1. Screening for OUD (average)**	**68%**
Use a screening protocol for OUD	95%
Use a standardized tool	22%
Screening done by clinical staff (physician, nurse, social worker, counselor)	87%
**2. Clinical assessment done by qualified treatment provider (average)**	**71%**
Clinical assessment done by clinical staff (physician, nurse, social worker, counselor)	71%
**3. Medically managed withdrawal (average)**	**88%**
Physician-approved protocol to address withdrawal from opioids	96%
Use FDA-approved medication for withdrawal management	81%
**4. MOUD administration (average)**	**64%**
Any MOUD available	92%
Buprenorphine available	73%
Methadone available	71%
Naltrexone available	73%
Available to anyone with OUD	20%
**5. MOUD for pregnant women (average) – limited to 174 jails with women**	**63%**
Any MOUD available to pregnant women	85%
Methadone available to pregnant women	72%
Buprenorphine available to pregnant women	66%
Methadone and buprenorphine available to pregnant women	53%
**6. Counseling and wrap-around services as part of MOUD (average)**	**80%**
Provide any other services as part of MOUD treatment	93%
Provide other substance use services/treatment as part of MOUD treatment	85%
Provide services for co-occurring disorders	59%
Provide self-help or other recovery support services	85%
**7. Collaborative relations with community MOUD providers (average)**	**61%**
Any of below	72%
Schedule appointments with MOUD provider in community	68%
Provide assistance completing intake paperwork for MOUD provider in community	60%
Facilitate exchange of key information with MOUD provider in the community	66%
Coordinate MOUD services with parole or probation	50%
**8. Assistance with applications for state Medicaid/insurance to pay for MOUD (average)**	**58%**
Any of below	73%
Jail staff assist with reactivating/applying for Medicaid or other types of insurance	61%
Jail staff help complete paperwork/application for Medicaid prior to release	58%
Jail has electronic access to submit Medicaid applications	36%
**9. Re-entry services (average)**	**38%**
Any of below	75%
Provide or arrange transportation to MOUD provider in community	39%
Provide transportation home	47%
Provide a bridge supply of multiple doses/days of MOUD	22%
Provide written prescriptions for MOUD	18%
Connect detainee to peer mentor/navigator/recovery coach	50%
Other things to facilitate linkage to MOUD	21%
**10. Overdose prevention (average)**	**68%**
Any of below	96%
Provide staff training on how to use Naloxone	93%
Provide staff with Naloxone kits to reverse overdose in jail	96%
Provide education and training to individuals while incarcerated on how to use Naloxone	33%
Provide individuals with Naloxone kits at release	30%

Specifically, 95% of the jails indicate the use of a protocol for OUD screening, yet only 22% utilize a standardized tool. Similarly, 96% of jails indicate they have a physician-approved protocol to address opioid withdrawal, however, fewer (81%) use an FDA-approved medication for withdrawal management. Most jails (92%) have some MOUD availability, and over 70% had each type of medication nominally “available,” however, only 20% stated they provide MOUD to anyone who is assessed with OUD. Most jails that house women have some type of MOUD available for pregnant women (85%); slightly over half (53%) have both methadone and buprenorphine available for pregnant individuals, with others providing either methadone or buprenorphine. Nearly all jails (93%) provide some type of counseling or wrap-around services along with MOUD, most frequently other non-MOUD treatment services and self-help groups or other recovery support (85% for each).

With regard to best practices at discharge, 72% of the jails indicate they engage in some collaborative activities with community MOUD providers or parole/probation to facilitate continuity of care, most often by scheduling appointments with a community MOUD provider (68%), and less often by coordinating services with parole or probation (50%). Most jails (73%) assist individuals with applying for state Medicaid or other insurance to ensure continuity of coverage, although the extent of these services is highly variable, with an average of 58% of items endorsed. Similarly, 75% of jails provide some re-entry services at discharge, including transportation at discharge (47%), written prescriptions for MOUD (18%), and other activities to facilitate linkage to MOUD (21%). Lastly, although most jails (96%) provide some type of overdose prevention services, these are mainly to provide training (93%) or Naloxone kits to staff (96%), with one third or fewer providing Naloxone education and training to individuals prior to their release or Naloxone kits at discharge.

### Association of OUD best practices availability with community and jail characteristics

We examined the association of OUD best practices availability, categorized as either high or low based on a median split, with jail and community characteristics (see Table 2). There were several differences between high and low best-practices groups with regard to community population characteristics. Jails in the high best-practices group had a higher mean that the low group in terms of total population (748,391 vs. 434,857), a higher proportion of Hispanic residents (13% vs. 8%), and a lower proportion of residents living in poverty (12% vs. 14%). Characteristics of jails also differed across high and low best-practice groups. Jails in the high group had lower average annual jail admissions (3682 vs. 5739) and lower average rates of jail admission per 100,000 population overall (296 vs. 347), as well as specifically for females (86 vs. 108), males (512 vs. 591), and white, non-Hispanics (219 vs. 267). There were no differences between best-practice groups, however, in census region, urbancity, or proximity to a MOUD provider, including by type of medication.

**Table 2:** Availability of Opioid Use Disorder Best Practices in Jails by Community and Jail Characteristics

	High (***n*** = 83)	Low (***n*** = 102)	Total (***n*** = 185)	Statistics	
**Community Characteristics**					
Census Region:				*X*^2^_(df = 3)_ = 3.73, *p* = 0.393	
Northeast	35 (42%)	16 (16%)	51 (28%)		
Midwest	17 (21%)	41 (40%)	58 (31%)		
South	19 (23%)	43 (42%)	62 (34%)		
West	12 (15%)	2 (2%)	14 (8%)		
Population Mean	748,391	434,857	575,553	**t**_**(df = 184)**_ **= 9.18,** ***p*** **= 0.002**	
(SD)	(966,485)	(544,702)	(776,912)		
[Median]	[469,116]	[220,186]	[268,539]		
Average % of Census Tracts:				*X*^2^_(df = 2)_ = 0.83, *p* = 0.660	
Urban	80%	69%	74%		
Suburban	15%	21%	19%		
Rural	4%	9%	7%		
Average % Race/ethnicity (not mutually exclusive)					
Hispanic or Latinx origin	13%	8%	11%	**X**^**2**^_**(df = 1)**_ **= 8.39,** ***p*** **= 0.004**	
White non-Hispanic	78%	79%	79%	*X*^2^_(df = 1)_ = 1.61, *p* = 0.205	
Black non-Hispanic	9%	13%	11%	*X*^2^_(df = 1)_ = 1.67, *p* = 0.196	
Average % below the poverty line	12%	14%	13%	**X**^**2**^_**(df = 1)**_ **= 13.60, p < 0.001**	
**Jail Characteristics**					
Total Jail Admissions Rate Mean	3682	5739	4816	**t**_**(df = 184)**_ **= 21.75,** ***p*** **< 0.001**	
(SD)	(2058)	(3160)	(2902)		
Total Jail Population Mean	1334	4890	1089	t_(df = 184)_ = 3.61, *p* = 0.055	
(SD)	(1868)	(1117)	(1513)		
**Jail/Community Rate Per 100,000 Mean (SD)**					
Total Population	296 (151)	347 (163)	324 (160)	**t**_**(df = 184)**_ **= 5.80,** ***p*** **= 0.016**	
Female	86 (62)	108 (79)	98 (72)	**t**_**(df = 184)**_ **= 4.40,** ***p*** **= 0.036**	
Male	512 (284)	591 (283)	555 (285)	**t**_**(df = 184)**_ **= 5.32,** ***p*** **= 0.021**	
Hispanic/Latinx	323 (341)	374 (492)	351 (430)	t_(df = 184)_ = 0.00, *p* = 0.959	
White non-Hispanic	219 (143)	267 (157)	246 (153)	**t**_**(df = 184)**_ **= 6.17,** ***p*** **= 0.013**	
Black non-Hispanic	1245 (1509)	1137 (826)	1185 (1180)	t_(df = 184)_ = 1.70, *p* = 0.680	
**Distance to MOUD Provider (< 10 miles from population center)**					
Any MOUD Provider	88%	87%	88%	*X*^2^_(df = 1)_ = 1.87, *p* = 0.171	
Buprenorphine Provider	87%	86%	87%	*X*^2^_(df = 1)_ = 2.12, *p* = 0.145	
Methadone Provider	59%	56%	58%	*X*^2^_(df = 1)_ = 1.39, *p* = 0.239	
Naltrexone Provider	78%	76%	77%	*X*^2^_(df = 1)_ = 2.79, *p* = 0.095	

### Training and resources needed to increase OUD best practices in jails

The interview assessed the types of training/technical assistance, resources, and education needed by respondents to increase their ability to provide OUD services (see Table 3). Needs for funding were most frequently endorsed, with respondents indicating they needed additional funds for medication (81%), clinical staff (80%), diversion prevention (76%), transportation to MOUD (65%), and MOUD in the community (61%). Needs for education were endorsed across a range of both correctional and community stakeholders; these include state/local politicians (68%), probation/parole staff (67%), general community (67%), correctional staff (65%), people who are incarcerated (65%), pregnant women (61%), judges (61%), and clinical staff/physicians (60%). The most frequently endorsed need pertaining to logistical support was help to minimize diversion (64%). Addressing stigma was another prominent concern, with 69% indicating a need for help in this area.Department of CorrectionsDOC administrators

**Table 3:** Training and Resources Needed to Expand MOUD in Jails and Facilitate Community Linkages

	Percent
**Additional funding needed for**	
Medication	81%
Clinical staff to administer and monitor MOUD	80%
Resources needed to prevent diversion	76%
Transportation to MOUD	65%
MOUD in the community	61%
**Education needed**	
State/local politicians and other key stakeholders	68%
Probation/Parole staff	67%
General community	67%
Correctional staff	65%
People who are incarcerated	65%
Pregnant women	61%
Judges	61%
Clinical staff/physicians	60%
Department of Corrections administrators	57%
District attorneys	55%
Churches	41%
Other	10%
**Help needed to address stigma and negative attitudes toward MOUD**	69%
**Needs inside jails**	
Logistical	
Minimize diversion	64%
Establish systems to screen people for OUD	57%
Become licensed opioid treatment provider	55%
Implement ECHO/Telemedicine	44%
Obtain waivers	43%
Test for illicit drug use	36%
Clinical	
Add medical staff	71%
Match needs with type of MOUD	62%
Switch between types of MOUD	60%
Supervise oral administration of MOUD	52%
Arrange dosing of methadone and/or buprenorphine by community program	49%
Administer, monitor, store medication	48%
Establish MOUD in pregnancy program	47%
MOUD administration	45%
**Re-entry support needs**	
Funding for MOUD post-release	70%
Same-day access to MOUD	69%
Solutions to regulatory, insurance, or managed care limits for post-release continuation of MOUD	69%
Access to sober housing	69%
Access to employment	65%
Provision of MOUD continuity of care upon re-entry into communities without MOUD	63%
Reactivation and/or application for Medicaid to help with re-entry	58%
Access to state identification	57%
Strategies for building community partnerships and establishing agreements for MOUD post-release	55%
MOUs for re-entry services	52%

With regard to clinical capacity, respondents indicated the highest needs for medical staff (71%), training on how to match client needs with type of medication (62%), and how to switch between types of medication (60%). Needs for re-entry support were also highly ranked by respondents; 70% indicated they needed additional funding for MOUD post-release; 69% endorsed the need for same-day access to MOUD; and 69% needed help to address barriers related to regulatory, insurance or managed care limits for post-release continuation of MOUD. Other highly rated needs to facilitate re-entry included access to sober/recovery housing (69%), employment (65%), and help for individuals who were returning to communities without MOUD availability (63%).

## Discussion

This study provides a comprehensive picture of the availability of best practices for addressing OUD among individuals in jails located in counties that have been most heavily impacted by the opioid epidemic. Given the high number and frequency of contacts that individuals with opioid use disorders have with jails (Zeng & Minton, 2021), it is essential that best practices are available to address the treatment needs of this population. Combined, these jails had over 3 million admissions during the 12-month reference period (2019) assessed in the interview. The counties in which they are located accounted for 66% of all the opioid-overdose deaths in the U.S. and had significantly higher rates of opioid-related overdose than the national average (20.3 vs 14.7 per 100,000 people) in the sampling referent period. Prior studies have evaluated the availability of evidence-based treatment practices for individuals with drug or alcohol use disorders generally in criminal justice settings (Friedmann et al., 2007); the current study focused on best practices specifically for individuals with OUD in these jails in areas heavily impacted by the opioid epidemic.

The study found that over 70% of jails in the sample provided some aspects of each of the ten best practice categories. When examined in terms of the extent of services provided within each category, however, there was considerable variability in implementation. It is noteworthy that several of the best practice areas that were least fully implemented pertained to continuity of care, including re-entry services at release, assistance with insurance applications, MOUD for pregnant people, and collaboration with community MOUD providers. Considerable research has documented the need to enhance collaborative relationships between jails and community MOUD providers to ensure continuity of care, as well as the challenges encountered in building these cross-system relationships (Friedmann et al., 2015; Monico & Mitchell, 2016; Welsh et al., 2015). Building these collaborations may require policy directives and dedicated funding to ensure that such collaborations can be developed and sustained.

Moreover, even if adequate re-entry access to community-based MOUD treatment is readily available, individuals released from jail face numerous challenges to treatment engagement (Mitchell et al., 2021). Access to Medicaid coverage for MOUD is essential, as it has been demonstrated to increase MOUD treatment utilization among individuals involved with the criminal justice system (Khatri et al., 2021). Several strategies to promote treatment linkage and engagement at jail re-entry have been examined, including peer and patient navigation interventions, intensive case management initiated while in jail and continuing following discharge, and motivational linkage and referral interventions (Grella et al., 2022). Studies participating in the National Institute on Drug Abuse (NIDA)-sponsored Justice Community Opioid Innovation Network (JCOIN) include several randomized clinical trials examining different linkage facilitation strategies to connect individuals with community-based MOUD treatment at jail discharge (Ducharme et al., 2021; Scott, Dennis, Grella, & Watson, 2021).

There was also variability in services availability by community-level characteristics. Availability of OUD best practices in jails was more common in counties that had larger populations, a higher percentage of Hispanic residents, fewer people living below the poverty line, and lower numbers/rates of jail admissions. Although we did not find differences by region or urbancity, the sample was small and may have limited power to detect such differences. Several studies have shown regional variation in OUD services, particularly related to rural and urban differences, that impact access to community MOUD treatment providers (Singer & Kopak, 2021). Thus, different strategies may be needed in rural areas with fewer community resources as compared with highly affected urban areas that are relatively better resourced. Moreover, successful MOUD implementation in jails is typically a lengthy process that requires a sustained commitment of funding, strong leadership, effective collaboration with community treatment providers, and use of data-driven strategies for continuous monitoring and quality improvement (Ferguson et al., 2019). Study respondents indicated high levels of need to educate diverse community stakeholders about MOUD provision in jail, which is a critical component of a multi-pronged strategy. A recent legislative initiative enacted in Massachusetts that established pilot programs in 5 counties to provide MOUD to individuals while in jail and at least 30 days prior to their release, including use of all 3 approved forms of MOUD, provides one example of legislative leadership in this area (see https://malegislature.gov/Laws/SessionLaws/Acts/2018/Chapter208).

Jails reported a number of challenges affecting their ability to expand availability of OUD best practices. The majority of jails indicate that additional funds were needed to help purchase and administer MOUD-related services, hire and train more clinical staff, or to prevent diversion. Over two-thirds also reported needing help to address stigma that often serves as an obstacle to services implementation, access, and retention. Most requested help educating an array of stakeholders about OUD and MOUD, including representatives of correctional, judicial, polictical, health care, and community sectors, all of whom can help to facilitate implementation of OUD-related services in jails.

### Implications for policy and practice

Given the high risks of relapse and opioid-overdose fatality following jail release (Alex et al., 2017), MOUD availability in the community, or the lack thereof, raises ethical and logistical issues for jails. Specifically, during the planning phase of this project, numerous stakeholders raised questions about the ethics of initiating MOUD with individuals while incarcerated, knowing that it was unlikely they would be able to continue their treatment upon release due to limited community access. As noted previously, community re-entry is a high-risk period for opioid relapse and overdose. Abruptly stopping treatment with MOUD is likely to bring on withdrawal and relapse to use, thus posing both ethical and safety concerns. In this study, several of the needs jail respondents identified were related to their inability to ensure continuity of MOUD treatment, which necessitates a broader, system-level approach to engage community MOUD treatment providers. This real concern has to be simultaneously addressed when pressing legislative guidance and/or in legal challenges to lack of MOUD provision related to the Americans with Disabilities Act, malpractice, or cruel and unusual punishment (Weizman et al., 2021).

The consequences of lack of MOUD availability, both during incarceration and upon release to the community, can be expressed in terms of potential opioid-related deaths that are averted when MOUD is provided to those who need it. This is illustrated in a recent simulation study using data from the National Center for Vital Statistics, which estimates that 668 lives out of every 10,000 incarcerated people nationally would be saved if all incarcerated individuals who had clinical need for MOUD had received it; additionally, 1609 lives out of every 10,000 incarcerated people would be saved if they had received MOUD both while incarcerated and after release (Macmadu et al., 2020). Achieving continuity of care across jail and community settings regarding MOUD service provision is key to addressing this public health crisis. Providing support for one but not both undermines the chances for success at mitigating the damaging effects of the continually evolving and expanding opioid epidemic (Jalal et al., 2018; Kertesz, 2017), which now accounts for more deaths in the United States than those from motor vehicle deaths, gun violence, and even exceeds deaths from human immunodeficiency virus (HIV) at the height of the 1990s HIV epidemic (Ciccarone, 2019).

As demonstrated by the wide range of needs identified by study respondents, jails need help particularly with funding to expand MOUD availability and clinical capacity; for re-entry support to ensure continuity of care, such as transportation to providers, funding for MOUD in the community, and same-day access to MOUD upon release; to address regulatory barriers; and to prevent diversion. It is noteworthy that a recent expert panel on state-level policies to improve access to OUD treatment identified automatic Medicaid enrollment for individuals leaving correctional settings as both highly implementable and effective in improving patient and population-level outcomes (Smart et al., 2022). In addition, study repondents identified MOUD-related stigma as a barrier to expansion of MOUD provision within local corrections and treatment systems and the need for education and training for stakeholders in both corrections and the local community to alleviate this barrier.

### Study strengths and limitations

The study has several strengths, including: a) focusing on jails most likely to implement OUD services because of the high needs in their communities stemming opioid overdose, b) a high jail response rate (76%) despite data collection occurring largely during the COVID-19 pandemic, which entailed considerable stress for these organizations (Stephenson, 2020), and c) detailed measures of services along the OUD service cascade overall that correspond with identified OUD best practices. In terms of limitations, this is an observational study at the organizational level based on jail reports from one or more jail officials. It does not include individual-level data from administrative records; thus, we are unable to determine receipt of OUD best practices at the individual level. As noted previously, for this reason we characterize the reported “availability” of OUD best practices, but cannot determine the extent of their actual provision. In addition, due to the selection criteria, study findings may not generalize to jails in areas less impacted by the opioid epidemic or that are in transition to increasing prevalence of individuals with OUD and opioid-related overdose. Non-respondent jails also tended to be from counties that were similar to those with lower rates of best practice implementation (i.e., less population, higher poverty levels, higher rates of incarceration). Thus, although the study achieved a relatively high response rate, availability of OUD best practices in jails may be even less than suggested by the study findings if non-responding jails were less likely to implement these practices. Conversely, the higher response rate of jails in more populous counties with lower rates of jail admissions per 100,000 people should be taken into consideration. Because of their higher numbers and/or population rates of opioid overdose, these areas may have more rapidly implemented aggressive policies to address their highly visible opioid-related problems, including expansion of OUD-related services in both community and correctional settings (Barocas et al., 2018; Clarke et al., 2018; Evans et al., 2021; Rawson et al., 2019; Simpatico, 2015).

We note that jail respondents often reported that some data were not available to answer questions, particularly the more detailed questions related to the number of people receiving specific services. For example, these include: number of persons confined on (target date) by type of offense, gender, pregnancy status, age, race, ethnicity; number that received medically supervised withdrawal for OUD and that received any MOUD and specific types of MOUD; and total number of new admissions and new admissions with OUD. Since these items had large amounts of missing data, they were dropped from the analyses, precluding analyses of the extent to which services were provided to the designated population of individuals with OUD, which is a limitation of the study. Nor could we independently audit or validate responses for those who provided this information.

It is important to note that this study is also just one wave. Although about half the interviews were completed during the first 10 months of the COVID-19 pandemic, the 2019 reference period occurred completely before the pandemic started. A subsequent survey will need to examine how availability of OUD best practices in jails has been impacted by the pandemic. Many of the resources consulted to identify OUD best practices were published after the study was initiated in 2017. Fortunately, many of these issues were identified in advance by representatives of these organizations who were advising the study design and instrumentation.

Finally, the study findings provide an indication of OUD service availability in jails at one moment in time, and policies in this area are rapidly changing. Future research is needed to track changes in service availability, particularly related to changes in opioid-related mortality, which has continued to steadily increase with greater use of synthetic opioids (e.g. fentanyl and fentanyl analogs) and combined opioid and stimulant use (Ciccarone, 2021). Future studies can build on these findings to develop optimal OUD service configurations and to refine OUD best-practice categories to reflect the relative importance/necessity of specific services and their relationship with outcomes.

## Conclusion

In sum, the study findings help to illuminate the extent to which OUD best practices have been implemented in jails that are most severely impacted by opioid overdose within their communities. It also highlights the significant need and interest from these jail in obtaining further training, technical assistance, education, and funding to expand the implementation of the OUD service cascade. Lastly, the study demonstrates the need for better coordination between jails and local communities to ensure continuity of MOUD treatment during incarceration and following release. To maximize public health and safety, policy experts, regulatory bodies, and governmental agencies need to consider the impact of the lost opportunities for reducing OUD, opioid-overdose deaths, and recidivism that result from the lack of greater MOUD availability and accessbility.

## Supplementary Information

**Additional file 1: Supplement A.** Maps of opioid overdose related deaths and rate per 100,000 by County.

**Additional file 2: Supplement B.** Instrument Development and Variable Domains for JCOIN Jail Interview.

**Additional file 3: Supplement C.** Analysis of Potential Non-Response Bias Among Targeted Jails.

## Data Availability

The datasets used and/or analyzed during the current study are available from the corresponding author on reasonable request.

## Abbreviations

COVID-19: Coronavirus disease
FDA: Food and Drug Administration
GAIN: Global Appraisal of Individual Needs
HIV: Human immunodeficiency virus
JCOIN: Justice Community Opioid Innovation Network
MOU: Memorandum of understanding
MOUD: Medication for opioid use disorder
NCCHC: National Commission on Correctional Health Care
NIDA: National Institute on Drug Abuse
OUD: Opioid use disorder
SAMHSA: Substance Abuse and Mental Health Services Administration
SPSS: Statistical Package for the Social Sciences
