# Supplementary material for: Availability of best practices for opioid use disorder in jails and related training and resource needs: findings from a national interview study of jails in heavily impacted counties in the U.S
Source: Health Justice. 2022 Dec 20;10:36. doi: 10.1186/s40352-022-00197-3 (PMC9763789; doi:10.1186/s40352-022-00197-3)
Supplement: Supplementary file 1 — Additional file 1: Supplement A. Maps of opioid overdose related deaths and rate per 100,000 by County. [file 40352_2022_197_MOESM1_ESM.docx]

**Supplement A: Maps of opioid overdose related deaths and rate per 100,000 by County**

*Figure A.1 Number of Opioid-Related Deaths by County: 2017*


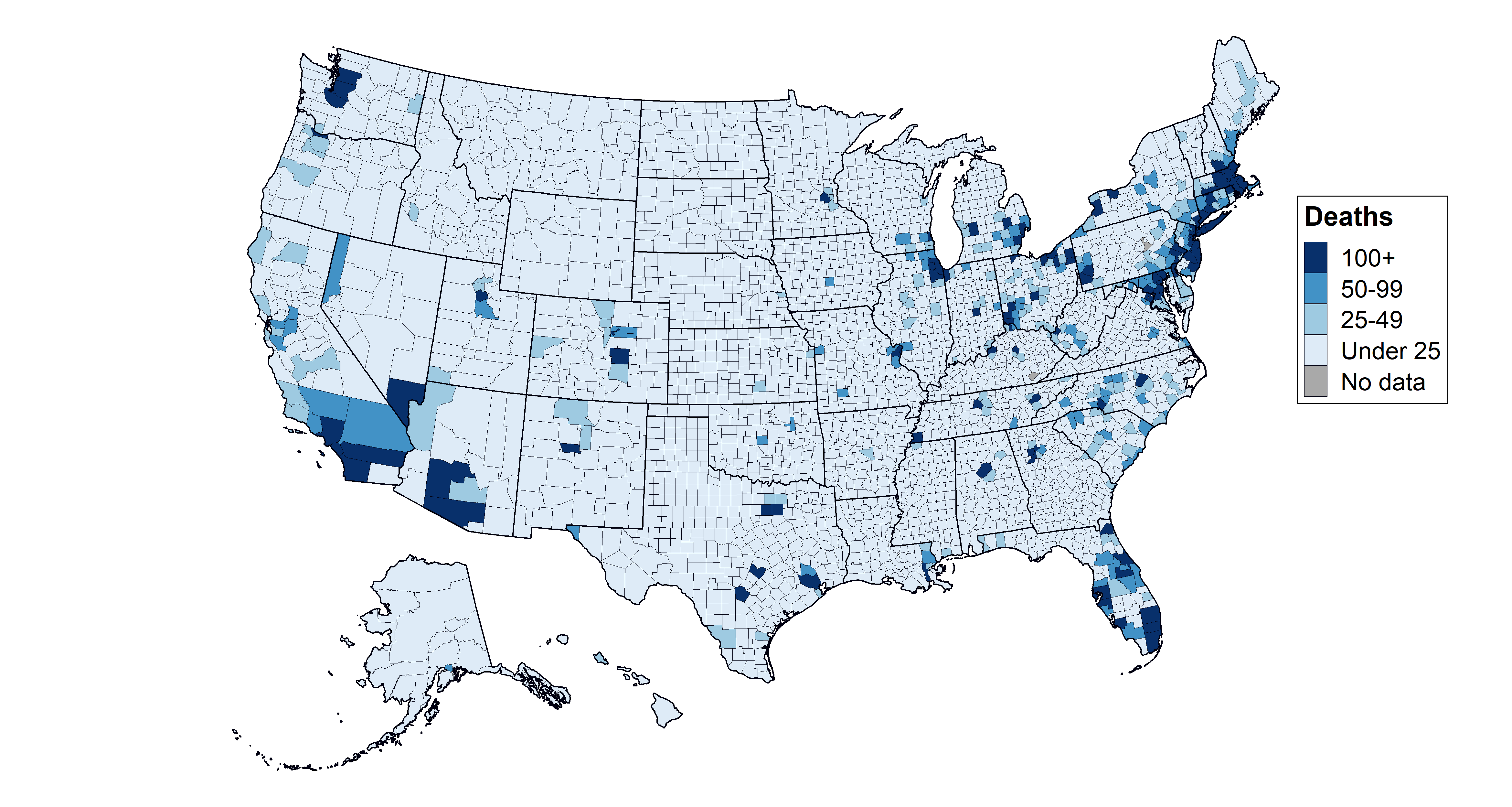
 *Figure A.2 Opioid-Related Death Rates by County: 2017*


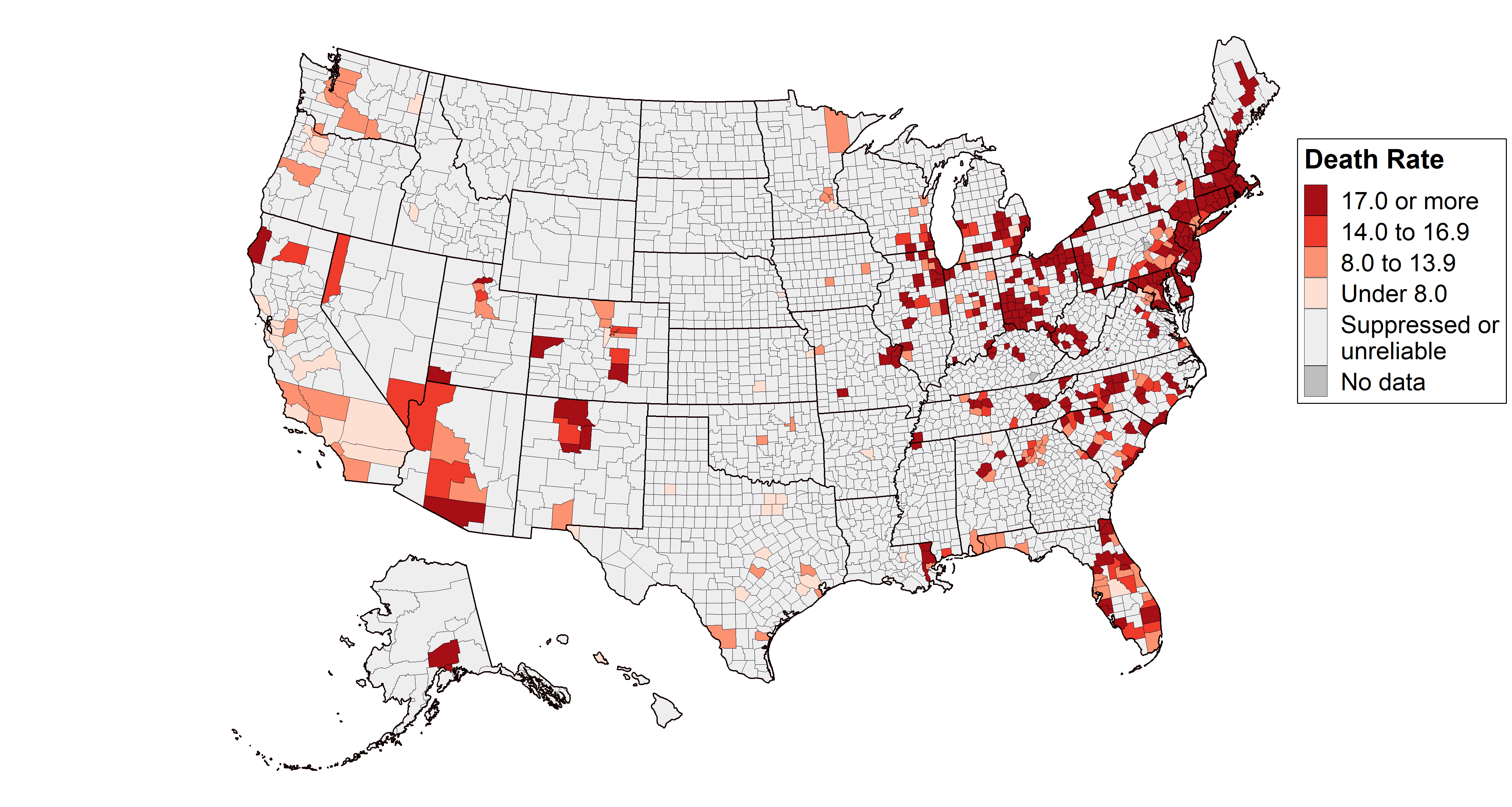


*Note*. Source: CDC 2017 multiple causes of death data.
