# Supplementary material for: Availability of best practices for opioid use disorder in jails and related training and resource needs: findings from a national interview study of jails in heavily impacted counties in the U.S
Source: Health Justice. 2022 Dec 20;10:36. doi: 10.1186/s40352-022-00197-3 (PMC9763789; doi:10.1186/s40352-022-00197-3)
Supplement: Supplementary file 2 — Additional file 2: Supplement B. Instrument Development and Variable Domains for JCOIN Jail Interview. [file 40352_2022_197_MOESM2_ESM.docx]

**Supplement B: Instrument Development and Variable Domains for JCOIN Jail Interview**

1. Instrument Development

To obtain background information and learn about current issues relevant to the development of the structured interview, the lead investigators attended multiple meetings and conferences for national associations involved with U.S. state and local correctional systems. They also reviewed prior surveys used by the national associations and other researchers, and solicited feedback from corrections staff, administrators, and high-ranking association members. Feedback on the interview content and structure was obtained from several individuals familiar with state correctional systems, including a Medication-Assisted Treatment Statewide Coordinator for the Department of Corrections (DOC) and the DOC Director of Psychiatry, Addiction, and Mental Health for another state. The interview was pretested with two individuals with relevant expertise to obtain feedback on the clarity, relevance, and flow of the questions.

Questions about medication for opioid use disorders (MOUD) focused on medications approved by the Federal Drug Administration (FDA) for treatment of OUD (i.e., methadone, buprenorphine, naltrexone), withdrawal management, and opioid-overdose reversal (i.e., naloxone). These questions used the generic names for medications, followed by the most common trade names in parentheses. Several descriptive questions used a “check all that apply” response format that addressed when, how, and by whom each of the medications was used.

2. Sources Consulted

The following survey instruments were reviewed:

- Illinois Criminal Justice Information Authority (ICJIA): *Survey on Opioid Medication-Assisted Treatment Use in Criminal Justice*
- National Sheriff’s Association: *Medication Assisted Treatment (MAT) in Jails Survey*
- Pew Charitable Trusts and Vera Institute of Justice: *Pew/Vera Survey of Correctional Health Care Services*
- U.S. Department of Justice, Bureau of Justice Statistics: *2014 Annual Survey of Jails*
- Pregnancy in Prison Statistics (PIPS) Study*: Treatment for Pregnant Women with Opioid Addiction in Jail Survey*.

The following resources were reviewed for best practices:

- *Expanding Access to Medications for Opioid Use Disorder in Corrections and Community Settings: A Roadmap for States to Reduce Opioid Use Disorder for People in the Justice System.* (Washington, DC: National Governors Association and American Correctional Association, January 2021). Accessed online at: <https://www.nga.org/center/publications/expanding-access-medications-oud-corrections-community-settings/>;
- Associated webinar: <https://www.nga.org/wp-content/uploads/2021/02/Roadmap-Release-Webinar-Slides-FINAL.pdf>
- *Jail-Based Medication-Assisted Treatment: Promising Practices, Guidelines, and Resources for the Field*. (National Sheriffs’ Association and the National Commission on Correctional Health Care, October 2018). Accessed online at: <https://www.ncchc.org/filebin/Resources/Jail-Based-MAT-PPG-web.pdf>
- *Medication-Assisted Treatment for Opioid Use Disorder in Correctional Settings (webinar).* GAINS Center for Behavioral Health and Justice Transformation, Substance Abuse and Mental Health Services Administration. Accessed online at: <https://www.youtube.com/watch?v=sMTUsJDr4G8>
- Sufrin, C., Beal, L., Clarke, J., Jones, R., & Mosher, W. D. (2019). Pregnancy outcomes in US prisons, 2016–2017. *American Journal of Public Health,* 109, 799-805.
- Sufrin, C., Sutherland, L., Beal, L., Terplan, M., Latkin, C. & Clarke, J. G. (2020). Opioid use disorder incidence and treatment among incarcerated pregnant women in the United States: Results from a national surveillance study. *Addiction*, 115, 2057-2065.

3. Variable Domains

The interview contained the following variable domains:

- Jail Characteristics: number of admissions in prior year, jurisdictional status (i.e., sheriff, county, other), accreditation status, health care delivery models used, lawsuits related to treatment of OUD with MOUD.
- Opioid Withdrawal Management: protocol for withdrawal management, duration of time the protocol has been in place, when protocol is implemented, screening tools for OUD withdrawal, medications used for medical management of opioid withdrawal, physician-approved protocol used, duration of time physician-approved protocol has been in place.
- Opioid Use Disorder (OUD) Screening: OUD screening protocol used, methods used to screen for OUD, OUD screening tools used, when screening is implemented, who conducts OUD screening.
- Medication for Opioid Use Disorders (MOUD): provision of any kind of MOUD; with regard to each form of MOUD (buprenorphine, methadone, naltrexone): duration that jail has provided MOUD, eligibility to receive MOUD, reason for using MOUD (i.e., continuity of care, induction and treatment during confinement, pre-release induction, to manage pain, other), method of MOUD administration, healthcare provider of MOUD, number of unique persons receiving MOUD; reasons for not providing each type of MOUD.
- MOUD for Pregnant Women: jail houses pregnant women, methods used for detoxification/withdrawal management and treatment for pregnant women with OUD, MOUD use following end of pregnancy.
- Other (Non-MOUD) Substance Use Treatment Services: outpatient SUD treatment, intensive outpatient SUD treatment, therapeutic community treatment, other recovery unit, transfer to residential/inpatient SUD in community, co-occurring SUD and mental health services, self-help meetings, other recovery support, other; forms of technology used to deliver SUD treatment or recovery support.
- Opioid Overdose Prevention: staff trainings on use of naloxone, duration of time staff training for naloxone has been provided, naloxone available for staff to use, naloxone kits provided to staff, staff carry naloxone kits, inmates are trained on how to use naloxone, duration of time naloxone training has been provided to inmates, inmates are provided with naloxone at release, duration of time that jail has provided naloxone kits to inmates at release; unique number of staff that received naloxone training, number of inmates that received naloxone kits and training prior to release, and number of naloxone kits provided to individuals at release; reasons for not providing naloxone in jail and not providing naloxone to individuals at release.
- Re-entry/Continuity of Care Services for MOUD: activities conducted in month prior to release regarding MOUD; activities conducted on day of release regarding MOUD; availability of MOUD in the month after release for former inmates; statements regarding use and availability of Medicaid for MOUD; time/distance to MOUD community provider at release.
- Technical Assistance Needs Related to the Provision of MOUD: need for education of various constituents, funding, staffing, services related to housing, employment; stigma-reduction; information on OUD and MOUD for pregnant women; diversion prevention; administration of MOUD; relationships with community MOUD providers; training for clinical staff; resources needed to establish linkages to MOUD in the community following release, including education, funding, regulatory, payment, Medicaid.
- Profile of People Confined in Jails (asked for number of persons confined on target date): type of offense, gender, pregnancy status, age, race, ethnicity, receiving medically supervised withdrawal for OUD, receiving any type of MOUD; total new admissions and new admissions with OUD, using MOUD, screen position for OUD; most common substances used by people prior to entering jail.
- Respondent Characteristics: age, gender, race, ethnicity, years in current position, years in corrections field.
- Respondent Comments or Suggestions
