# Supplementary material for: Availability of best practices for opioid use disorder in jails and related training and resource needs: findings from a national interview study of jails in heavily impacted counties in the U.S
Source: Health Justice. 2022 Dec 20;10:36. doi: 10.1186/s40352-022-00197-3 (PMC9763789; doi:10.1186/s40352-022-00197-3)
Supplement: Supplementary file 3 — Additional file 3: Supplement C. Analysis of Potential Non-Response Bias Among Targeted Jails. [file 40352_2022_197_MOESM3_ESM.docx]

| **Supplement C: Analysis of Potential Non-Response Bias Among Targeted Jails** | | | | | |
| --- | --- | --- | --- | --- | --- |
|  | **Total**  **(n=250 jails)** | **Respondents (n=185)** | **Non-Respondents (n=65)** | **Statistics** | |
| **COMMUNITY CHARACTERISTICS** | | | | | |
| Census Region: |  |  |  | *X*^2^_(df=3)_=1.49, p=0.685 | |
| Northeast | 24% | 28% | 12% |  |  |
| Midwest | 30% | 31% | 28% |  |  |
| South | 39% | 34% | 54% |  |  |
| West | 7% | 8% | 6% |  |  |
| Population Mean  (SD) | 560,310  (939,485) | 575,523 (776,912) | 517,011 (1,302,341) | **t_(df=249)_= 9.44, p=0.002** | |
| Average % of Census Tracts: |  |  |  | *X*^2^_(df=3)_=0.89, p=0.640 | |
| Urban | 11% | 11% | 11% |  |  |
| Suburban | 78% | 79% | 77% |  |  |
| Rural | 12% | 11% | 13% |  |  |
| Average % Race/ethnicity (not mutually exclusive) | | | |  | |
| Hispanic or Latinx origin | 13% | 8% | 11% | *X*^2^_(df=1)_=0.70, p=0.404 | |
| White non-Hispanic | 78% | 79% | 79% | *X*^2^_(df=1)_=0.07, p=0.790 | |
| Black non-Hispanic | 9% | 13% | 11% | *X*^2^_(df=1)_=0.29,p=0.589 | |
| Average % below the poverty line | 14% | 13% | 15% | *X*^2^_(df=1)_=4.01, p=0.045 | |
| **JAIL DATA** | | | | |  |
| Total Jail Admissions Rate Mean  (SD) | 5,047  (3,191) | 4,816  (2,902) | 5,713  (3859) | t_(df=249)_= 1.92, p=0.166 | |
| Total Jail Population Mean  (SD) | 1,083  (1,790) | 1,086  (1,517) | 1,075  (2,425) | t_(df=249)_= 3.30, p=0.069 | |
| **JAIL/COMMUNITY RATE PER 100,000 MEAN (SD)** | | | | | |
| Total Population | 346 (180) | 324 (160) | 409 (217) | **t_(df=249)_= 7.96, p=0.005** | |
| Female | 106 (76) | 98 (73) | 129 (83) | **t_(df=249)_= 8.03, p=0.005** | |
| Male | 592 (317) | 555 (285) | 699 (377) | **t_(df=249)_= 7.53, p=0.006** | |
| Hispanic/Latinx | 370(524) | 351 (430) | 425(731) | t_(df=249)_= 0.19, p=0.661 | |
| White non-Hispanic | 264 (166) | 246 (153) | 317 (193) | **t_(df=249)_= 8.41, p=0.004** | |
| Black non-Hispanic | 1,292 (1,368) | 1,185 (1,180) | 1,601(1,780) | t_(df=249)_= 2.24, p=0.134 | |
| **Availability of MOUD provider (<10 miles from population center)** | | | | |  |
| Any MOUD provider | 88% | 88% | 89% | X^2^_(df=1)_=1.50, p=0.220 | |
| Buprenorphine provider | 87% | 87% | 87% | X^2^_(df=1)_=1.90, p=0.343 | |
| Methadone provider | 58% | 58% | 60% | X^2^_(df=1)_=0.56, p=0.456 | |
| Naltrexone provider | 77% | 77% | 78% | X^2^_(df=1)_=0.53, p=0.470 | |
